# Utilization of public or private health care providers by febrile children after user fee removal in Uganda

**DOI:** 10.1186/1475-2875-8-45

**Published:** 2009-03-14

**Authors:** Elizeus Rutebemberwa, George Pariyo, Stefan Peterson, Goran Tomson, Karin Kallander

**Affiliations:** 1 Department of Health Policy Planning and Management, Makerere University School of Public Health, PO Box 7072, Kampala, Uganda; 2 Division of International Health, IHCAR, Department of Public Health Sciences, Karolinska Institutet, SE 17177 Stockholm, Sweden; 3 International Maternal and Child Health Unit, Uppsala University, Sweden; 4 Department of Epidemiology and Biostatistics, Makerere University School of Public Health, PO Box 7072, Kampala, Uganda

## Abstract

**Background:**

Despite investments in providing free government health services in Uganda, many caretakers still seek treatment from the drug shops/private clinics. The study aimed to assess determinants for use of government facilities or drug shops/private clinics for febrile illnesses in children under five.

**Methods:**

Structured questionnaires were administered to caretakers in 1078 randomly selected households in the Iganga – Mayuge Demographic Surveillance site. Those with children who had had fever in the previous two weeks and who had sought care from outside the home were interviewed on presenting symptoms and why they chose the provider they went to. Symptoms children presented with and reasons for seeking care from government facilities were compared with those of drug shops/private clinics.

**Results:**

Of those who sought care outside the home, 62.7% (286/456) had first gone to drug shops/private clinics and 33.1% (151/456) first went to government facilities. Predictors of having gone to government facilities with a febrile child were child presenting with vomiting (OR 2.07; 95% CI 1.10 – 3.89) and perceiving that the health providers were qualified (OR 10.32; 95% CI 5.84 – 18.26) or experienced (OR 1.93; 95% CI 1.07 – 3.48). Those who took the febrile child to drug shops/private clinics did so because they were going there to get first aid (OR 0.20; 95% CI 0.08 – 0.52).

**Conclusion:**

Private providers offer 'first aid' to caretakers with febrile children. Government financial assistance to health care providers should not stop at government facilities. Multi-faceted interventions in the private sector and implementation of community case management of febrile children through community medicine distributors could increase the proportion of children who access quality care promptly.

## Background

Infant and child mortality rates due to febrile illnesses are high in resource poor countries, especially in sub-Saharan Africa [1]. With the millennium development goal number four, many countries have targeted to reduce under-five mortality of the 1990 level by two thirds by 2015 [2]. In Uganda, there is a high disease burden from febrile illnesses with malaria contributing 30 – 50% of outpatient burden and 35% of hospital admissions [3]. Those affected by malaria are mostly women and children under five years. Much of the effort at health facility level, has been to improve quality and reduce costs of care in government facilities [4]. In 2001, the government removed user fees from all the government health facilities except for the private wing in the district hospitals and there was a rapid increase in utilization of care [5]. Studies have demonstrated that even after removal of user fees quality of care was maintained [6].

However, user fees in government facilities is not the only barrier to health care utilization [7,8]. The initial increase in health facility utilization following the abolition of user fees in Uganda in 2001 has levelled off since the 2004/05 financial year [3]. Studies from other countries also indicate that after removal of user fees, there is an initial increase in new clients but again starts falling after some time [9]. Most of the febrile children receive their first treatment from the private sector, which, in some places, attend to more outpatients than the government facilities [10]. Most drug shops/private clinics in Uganda are unregistered and some of them are run by unqualified staff who give poor quality care [11]. It is unclear what factors drive caretakers to choose to pay for poor quality services at drug shops/private clinics instead of seeking free care from government facilities.

Caretakers' choice of care provider is often an interplay of many factors [12]. Affordable and available drugs [12,13], geographical accessibility [14] and appropriate opening hours [15,16] are important contributors to caretakers' choice. Other factors include travel time, education, age, sex, quality of care [17] education and household size [18]. Factors leading to preferences for private providers in circumstances where government has removed user fees have not been well explored. The aim of this study was to quantitatively assess reasons why caretakers of children under five utilize government facilities or drug shops/private clinics for treatment of febrile illness.

## Methods

### Study area and population

The study was conducted in the Iganga/Mayuge Demographic Surveillance Site (DSS), which has a population of about 67,000 people in about 13,000 households. The DSS is located on the boundary between the districts of Iganga and Mayuge, about 115 km from the capital Kampala. The area is predominantly rural with only about 10% living in a sub-urban environment. The major tribe is the Basoga and most people depend on subsistence agriculture engaging mainly in food crops.

The DSS area has one district hospital, four government Health Centre IIIs, five government Health Centre IIs and three Non Governmental Organization (NGO) Health Centre IIs. The health system in Uganda is arranged in a hierarchical system starting with the Health Centre I (HC I) at the village level which acts as an outpost for outreach services going up to the National Referral Hospitals with advanced tertiary care (Table 1). Health Centre IIs and IIIs mainly provide outpatient primary health care. The district hospital, one government Health Centre III and one government Health Centre II are located in Iganga town. The remaining government health facilities and all the NGO facilities are scattered in the rural part of the DSS. None of the Health Centre IIIs in the DSS has a functional laboratory. All the government and NGO facilities have clinical officers or nurses for health care delivery apart from the hospital, which, in addition has doctors.

**Table 1:** The structure of the Uganda National Health system

Health unit	Services	Location	Population
Health Centre I	Outpost for outreach services	village	1,000

Health Centre II	Out patient services only	Parish	5,000

Health Centre III	Out patient services, Maternity, General Ward and laboratory	Sub-county	20,000

Health Centre IV	Outpatients, Wards, Theatre, Laboratory and blood transfusion	County	100,000

General Hospital	General Hospital care, Secondary services, laboratory and X-ray	District	100,000 – 1,000,000

Regional Referral Hospital	Specialists services	Region (3 – 5 districts)	1,000,000 – 2,000,000

National Referral Hospital	Advanced Tertiary Care	National	Over 20,000,000

Adapted from Government of Uganda, Health Sector Strategic Plan I, 2000/01 – 2004/05

Other care providers include 122 drug shops/private clinics, which are mostly located in the trading centres in the rural areas and some in Iganga town. In April 2007, an inventory of these private drug outlets was done by the DSS. There were 74 variety shops, which sell commodities like sugar, groceries and stationery in addition to the drugs. Most of the drugs found in these variety stores are pain killers, such as paracetamol, and cheap antimalarials, such as chloroquine. There were also six private clinics that sell drugs and can give treatment through injections and 42 drug shops, which mainly sell drugs. Like in many other parts of Uganda, the owners of these drug shops and private clinics may be qualified but on the day-to-day basis, some of them are run by staff without any formal training in health care. The drugs they provide are often of poor quality [11]. There are some community medicine distributors (CMDs) who are scattered in the villages but they are not very active since the change of first-line of treatment from chloroquine and sulphadoxine/pyrimethamine to arthemether/lumefantrine (Coartem^®^). Coartem has not yet been distributed at the community level. There are some traditional healers and spiritualists scattered in the villages and the town but the majority of the patients seek care from the biomedical providers [10].

The study population consisted of caretakers residing in the DSS and who had an under five child that had fallen sick with a febrile illness within the previous two weeks. The child had to be present during the interview to assist the caretaker in recalling the illness episode. All the children whose care seeking behaviour was investigated had presented with fever with or without any other symptom.

### Sampling and data collection

During a DSS update round in January–February 2007, 1,415 households with children under five were selected using simple random sampling. Based on the assumption that 52% of the children had had fever in the previous two weeks [19], that 60% would have sought care from outside the home [20] and accounting for 15% drop out, a minimum sample size of 1,415 children was needed to determine the proportion who had sought care outside the home, using 5% absolute precision and 95% confidence. The DSS field assistants dedicated the first three weeks of the update round to visit the selected households and collected data for this study. The field assistants were trained on the study tool by the first author, which was then translated into the local language, then pre-tested and adjustments made. During the household visits the primary caretaker was asked if any of the children under five years of age had fallen ill in the previous two weeks. If two or more children had been sick, one was randomly selected using the ballot method. For the selected child, the caretaker was asked about presenting symptoms, whether care had been sought outside the home, which health provider they consulted and reasons for choosing the particular provider.

The reasons for going to a provider were explored by reading a list of alternatives which had been adapted from the quality dimensions used in Guinea [21] and Burkina Faso [22,23] and which had been piloted in the DSS through 4 focus group discussions with mothers of children below five years. The final list of reasons assessed for having gone to a certain health care provider included the conduct, qualifications and experience of the health provider, provider being polite, having 'cheap' treatment, giving treatment on credit, being near, giving first aid treatment, having access to good equipment or infrastructure, or being a place where the caretaker could easily get subsequent treatment for the child. The reasons were read out and caretakers were asked to respond 'Yes' or 'No' to whether the reason had been considered when deciding where to go for care. For households where the caretaker was absent during the household visit, three more attempts were made before the household was coded as lost-to-follow up.

### Data management and analysis

The questionnaires were collected at the DSS headquarters on a daily basis and checked for consistency by the first author and the DSS staff. Data was entered using FoxPro computer package, cleaned, and afterwards linked with the DSS database to get socio-demographic variables like the household head education and household socio-economic status. It was then exported to STATA 10 (STATA Corporation, College Station, TX, USA) for bivariate and multivariate analysis. The calculation of socio-economic status has been described previously by Rutebemberwa *et al *[24]. The main presenting symptoms of children who were taken outside and reasons caretakers gave for having gone to government or private providers were identified. Those who went to government were compared with those who went to the drug shops and private clinics. Government facilities are ideally expected to provide free treatment to patients [4]. During the piloting of the variables in focus group discussions, the mothers of children under five years were not differentiating a private clinic from a drug shop possibly because they receive similar services from them. They could speak of a clinic as a drug shop and a drug shop as a clinic. Consequently, the drug shops and private clinics were grouped together because of close similarity in practices, similar to what was reported by Tawfik *et al *that many drug shops provide clinical management and clinics sell drugs to clients [11].

Analysis was done at bivariate level and all variables with a *p*-value of less than 0.1 qualified for the multivariable logistic regression model for predictors of utilization of government facilities or drug shops/private clinics.

### Ethical approval

The study was approved by the Institutional Review Board of Makerere University School of Public Health and the Uganda National Council for Science and Technology (Ref. HS 72). Permission was granted by the DSS management and the village local council leaders to conduct the study. Verbal consent was received from all the respondents.

## Results

Of the 1,415 households selected from the DSS database, data was collected from 1,078 (76.2%) of the households. Of the 377 households where data was not collected, 303 families had migrated. Another 26 households had no under-five child at the time of the survey. In the remaining eight households, the children were not at home at the time the research team went there. Comparing those who had migrated with those we found at home, there was no significant difference with respect to household head education status (chi square 5.49, 4 degrees of freedom, p-value 0.241) and household socio-economic status (chi square 8.69, 4 degrees of freedom, p-value 0.069). Out of the 1,078 households visited, 793 (73.6%) had at least one child who had been sick in the previous two weeks. Of the children who had been sick, 759 (95.7%) had presented with fever and 456 (60.1%) of these had sought care outside the home: 95.8% (437/456) from government facilities or drug shops/private clinics.

The main providers of treatment for febrile children were drug shops/private clinics (62.7%; 286/456) and government health facilities (33.1%; 151/456). Very few had gone to Community Medicine Distributors (CMDs) (2.0%; 9/456), NGOs (1.5%; 7/456) or to neighbours and other categories (0.7%; 3/456). There were no significant differences between age, sex and education of the household head, age and sex of the children and socio-economic status of the households that had gone to the drug shops/private clinics and those who went to government facilities (Table 2).

**Table 2:** Socio-demographic characteristics of household heads, children and the socio-economic status of households

**Characteristic**	**Government**	**Drug shops/private clinics**	**OR (95% CI)**	**p-value**
**1. Age of household head**	**n = 151 (%)**	**n = 286 (%)**		

< 29	39 (25.8)	63 (22.0)	1	

30 – 39	57 (37.8)	121 (42.3)	0.76 (0.46 – 1.27)	0.293

40 – 49	32 (21.2)	63 (22.0)	0.82 (0.46 – 1.47)	0.507

≥ 50	23 (15.2)	39 (13.6)	0.95 (0.50 – 1.83)	0.884

**2. Sex of household head**	**n = 151 (%)**	**n = 286 (%)**		

Female	17 (11.3)	40 (14.0)	1	

Male	134 (88.7)	246 (86.0)	0.78 (0.43 – 1.43)	0.421

**3. Education of household head**	**n = 140 (%)**	**n = 266 (%)**		

None	17 (12.1)	30 (11.3)	1	

Primary 1 – 4	31 (22.1)	60 (22.6)	0.91 (0.44 – 1.91)	0.806

Primary 5 – 7	54 (38.6)	112 (42.1)	0.85 (0.43 – 1.68)	0.641

Senior 1 – 4	29 (20.7)	57 (21.4)	0.90 (0.43 – 1.90)	0.777

Senior 5 – 6	9 (6.4)	7 (2.6)	2.27 (0.70 – 7.39)	0.162

**4. Age of child**	**n = 151 (%)**	**n = 286 (%)**		

≤ 11 months	41(27.2)	67 (23.4)	1	

12 – 35 months	72 (47.7)	135 (47.2)	0.87 (0.54 – 1.41)	0.577

36 – 59 months	38 (25.2)	84 (29.4)	0.74 (0.43 – 1.28)	0.278

**5. Sex of child**	**n = 151 (%)**	**n = 286 (%)**		

Female	79 (52.3)	139 (48.6)	1	

Male	72 (47.7)	147 (51.4)	0.81 (0.55 – 1.20)	0.295

**6. Socio-economic status of the household**	**n = 133 (%)**	**n = 267 (%)**		

1^st ^quintile	29 (21.8)	58 (21.7)	1	

2^nd ^quintile	31 (23.3)	70 (26.2)	0.88 (0.48 – 1.64)	0.699

3^rd ^quintile	32 (24.1)	57 (21.4)	1.12 (0.60 – 2.09)	0.716

4^th ^quintile	18 (13.5)	47 (17.6)	0.77 (0.38 – 1.55)	0.458

5^th ^quintile	23 (17.3)	35 (13.1)	1.31 (0.66 – 2.63)	0.438

Having a running nose was the main additional symptom febrile children presented with in both the government facilities (69.5%; 105/151) and the drug shops/private clinics (71.7%; 205/286). The second major additional symptom was cough for government facilities (55.0%; 83/151) as well as for drug shops/private clinics (48.3%; 138/286). The caretakers who sought care from government facilities went there because they perceived the provider to be qualified (56.6%; 90/151), nearby (45.0%; 68/151), or experienced (43.3%; 65/151). The main reasons cited for caretakers seeking treatment from drug shops/private clinics were because the provider was nearby (71.9%; 205/286), treatment was considered cheap (30.9%; 88/286) or they could get treatment on credit (27%; 77/286).

In the bivariate analysis, children who had been taken to government facilities were more likely to have presented with diarrhoea (OR 1.57; 95% CI 1.01 – 2.44) or with vomiting (OR 1.69; 95% CI 1.07 – 2.67). Caretakers also sought treatment from government facilities because these facilities were perceived to be more likely to have qualified (OR 14.10; 95% CI 7.67 – 25.92) and experienced (OR 3.59; 95% CI 2.26 – 5.71) health providers. Other reasons given were the presence of good equipment (OR 4.92; 95% CI 2.74 – 8.81) and good infrastructure (OR 2.96; 95% CI 1.43 – 6.09). Those who went to drug shops/private clinics for treatment were more likely to have gone there because the provider was near (OR 0.32; 95% CI 0.21 – 0.49) or the treatment was a form of first aid (OR 0.32; 0.15 – 0.68) (Table 3).

**Table 3:** Symptoms children presented with and reasons caretakers gave for having used government or private providers

**Variable**	**Government**	**Drug shops & private clinics**	**Crude** **OR (95% CI)**	**p-value**	**Adjusted** **OR (95% CI)**	**p-value**
Presenting symptom *	**n = 151 (%)**	**n = 286 (%)**				

Cough	83 (55.0)	138 (48.3)	1.31 (0.88 – 1.95)	0.182	N/A	

Running nose	105 (69.5)	205 (71.7)	0.90 (0.59 – 1.39)	0.640	N/A	

Difficulty in breathing	30 (19.9)	62 (21.7)	0.90 (0.55 – 1.46)	0.660	N/A	

Fast breathing	18 (11.9)	52 (18.2)	0.61 (0.34 – 1.09)	0.090	0.67 (0.32 – 1.40)	0.284

Convulsions	9 (6.0)	22 (7.7)	0.76 (0.34 – 1.70)	0.503	N/A	

Pallor	4 (2.7)	11 (3.9)	0.68 (0.21 – 2.18)	0.514	N/A	

Diarrhoea	49 (32.5)	67 (23.4)	1.57 (1.01 – 2.44)	0.042 †	0.98 (0.54 – 1.79)	0.947

Vomiting	44 (29.1)	56 (19.6)	1.69 (1.07 – 2.67)	0.024 †	2.07 (1.10 – 3.89)	0.024 ‡

Perception of provider*	**n = 151 (%)**	**n = 286 (%)**				

Conduct of health provider	51 (33.8)	53 (18.6)	2.23 (1.41 – 3.53)	<0.001 †	1.70 (0.94 – 3.08)	0.077

Qualification of health provider	90 (56.6)	27 (9.5)	14.10 (7.67 – 25.92)	<0.001 †	10.32 (5.84 – 18.26)	<0.001 ‡

Experience of health provider	65 (43.3)	50 (17.5)	3.59 (2.26 – 5.71)	<0.001 †	1.93 (1.07 – 3.48)	0.030 ‡

Provider is polite	6 (4.0)	23 (8.1)	0.47 (0.19 – 1.19)	0.103	N/A	

Good equipment	44 (29.1)	22 (7.7)	4.92 (2.74 – 8.81)	<0.001 †	1.88 (0.88 – 4.02)	0.103

Good infrastructure	20 (13.3)	14 (4.9)	2.96 (1.43 – 6.09)	0.002 †	1.31 (0.50 – 3.43)	0.576

Treatment being cheap	58 (38.4)	88 (30.9)	1.40 (0.92 – 2.11)	0.113	N/A	

Provider is nearby	68 (45.0)	205 (71.9)	0.32 (0.21 – 0.49)	<0.001 †	0.62 (0.36 – 1.06)	0.080

Easier to get more treatment there	37 (24.5)	58 (20.4)	1.27 (0.79 – 2.03)	0.318	N/A	

To get first aid	9 (6.0)	47 (16.5)	0.32 (0.15 – 0.68)	0.002 †	0.20 (0.08 – 0.52)	0.001 ‡

The reference category was the government facilities.

* Multiple answers were given for the presenting symptom or perception of provider.

† Significant at bivariate analysis.

‡ Significant at multivariable analysis.

N/A stands for Not Applicable. It is for those variables whose p ≥ 1 and were hence not included in the multivariable analysis.

In the multivariable analysis, child having presented with vomiting (OR 2.07; 95% CI 1.10 – 3.89) and provider having good qualification (OR 10.32; 95% CI 5.84 – 18.26) or experience (OR 1.93; 95% CI 1.07 – 3.48) were the variables that remained significantly associated with having used government facilities. An independent predictor for using drug shops/private clinics was having gone there for first aid (OR 0.20; 95% CI 0.08 – 0.52) (Table 3).

## Discussion

Despite no official user fees at government facilities, 2/3 of children with fever were taken to drug shops/private clinics as the first source of care outside the home. A big proportion of the caretakers who sought care from drug shops/private clinics went there because these providers were perceived to be near, treatment was perceived to be cheap and they could get treatment on credit. Getting 'first aid' was an independent predictor of having sought health care from drug shops/private clinics. Caretakers who went to government facilities perceived health workers in these facilities to be qualified and experienced. The children were also more likely to have presented with vomiting.

The finding that caretakers with febrile children seek care from sources where they can obtain 'first aid' has been highlighted previously in Uganda [25], where anti-malarial treatment from Community Medicine Distributors (CMDs) was utilized as a form of 'first aid'. From the pilot focus group discussions, 'first aid' was described as the first treatment given to the child while the condition is being monitored and if the condition worsens the caretaker proceeds to a higher-level facility. Patients in Uganda and Kenya sought care from drug shops/private clinics after home treatment had failed, suggesting that the private providers fill in the gap between home treatment and government health care [26,27]. Drug shops and private clinics are the most common formal providers available in rural areas [10]. Government facilities charging unofficial fees and patients buying cheap drugs from drug shops, even if they are only pain killers, could also partly explain the high utilization of drug shops and private clinics. If the quality and coverage of basic primary health care is to improve, the services actually used by the population need to improve. Hence the government's support should not end at the public health facilities but need to also include the private sector that often provides the first treatment outside the home. This could be done alongside the Home Based Management of Fever using community medicine distributors, who are now to receive Coartem for distribution at community level.

The study found that caretakers used drug shops and private clinics because these providers were perceived to be near, to be cheap and because of the possibility of getting treatment on credit. Treatment on credit probably reflects the inability of many caretakers to raise cash at the time of illness for treatment and/or for travel. This is similar to findings from studies in Ghana [17]. Removing or subsidizing user fees does not necessarily make health care affordable to caretakers as other costs, such as for transport and loss of working time, can add up to 79% of the total cost of seeking treatment [28]. Abolishing user fees in Uganda did not reduce these other direct or indirect costs. Mothers of children under five are especially vulnerable to suffer from these additional costs, as women's lack of control over household resources is well documented [29,30]. Buying treatment on credit from the drug shops/private clinics may be a way of enabling mothers without cash to access treatment which they would pay for later when they get the funds.

In this study, those who went to government health facilities did so because of expecting to be handled by qualified and experienced health providers. Seeking care from government facilities because of having qualified workers [3] is similar to findings from other studies done in Uganda, Kenya and Ghana [10,31,32]. It could also be a reflection of the progress in the implementation of the integrated management of childhood illness (IMCI) programme. By 2007, the proportion of sick under fives in Uganda seen by a health worker using IMCI guidelines stood at 60% [3]. However, unlike some studies which indicated that caretakers perceived drug shops and private clinics to provide quality care [23,33,34], the respondents did not attribute good infrastructure or good equipment to the private providers. It has been documented that most Ugandan shops/private clinics are run by unqualified staff who often use medicines of poor quality [11]. A recent survey showed that the malaria medicines found in the private sector commonly was of types with lower efficacy while first-line treatment Coartem, which has a very high private market price, was rarely available outside the public sector. With the effective artemisinin combination therapy not being easily available in drug shops, caretakers may be using ineffective non-artemisinin therapies, such as chloroquine and sulphadoxine-pyrimethamine, that are 5 – 10 times cheaper for a child of five years [35]. Thus, the majority of febrile children who are taken for care to drug shops/private clinics are not likely to receive effective malaria treatment.

One approach to increase access to high quality, pre-packaged drugs for malaria, which is currently piloted in a number of districts in Uganda, is to subsidize Coartem in drug shops and private clinics and train their attendants in malaria case management [35] Training alone may not be enough. Experiences where multifaceted interventions with drug shops' owners and private clinic providers has increased the provision of quality care have been documented in Uganda, Vietnam, India and Pakistan [11,36-39]. In Kenya it resulted in increased proportion of children getting proper malaria treatment [27]. The combination of private provider training with the subsidization of quality drugs in the private sector, is in line with the policy for public-private partnership and could be a way of meeting the community demand for affordable and appropriate treatment at a source and level where most caretakers seek care. However, caution has been raised that these private providers may sell the drugs to other vendors [16], and close monitoring of their performance would be necessary to ensure that the subsidized drugs actually reach the sick children [40]. The first treatment outside the home is important if children are to get prompt and appropriate treatment. Differences occur between countries and even within the same country as to which provider is easily accessible to the caretakers of children under five. While in The Gambia, government facilities are the dominant first option [41], in this study, it is the private providers. It is imperative that local conditions need to be evaluated before a policy of providing cheap treatment through public or private provider is taken.

Another approach to enable children under five to access prompt, adequate and affordable treatment for febrile illnesses is through integrated community case management of illnesses, such as malaria, pneumonia and diarrhoea [42,43]. In 2002, Uganda instituted the Home Based Management of Fever program (HBMF) and deployed Community Medicine Distributors (CMDs) to distribute antimalarials. However, very few caretakers stated having sought care from CMDs, likely due to the fact that HBMF in effect came to a halt after the 2004 change in the anti-malarial first-line treatment policy, moving from a combination of chloroquine + sulphadoxine/pyrimethamine (SP) to arthemether/lumefantrine combination (Coartem). While the old drug was discouraged the new one was not availed to the CMDs. Other studies have shown that low utilization of CMDs results from their inability to provide treatment for other conditions, such as Oral Rehydration Salt (ORS) for diarrhoea and vomiting [25]. A child with diarrhoea or vomiting can quickly deteriorate and to avoid dehydration, the use of ORS is necessary but is usually not available in the private sector. This study confirmed that febrile children with vomiting were more likely to have been taken to government facilities, which has also been documented in another Ugandan study where 94% of children with diarrhoea and vomiting went to the government health facilities [10]. Hence, making ORS available with CMDs could contribute to the reduction of morbidity and mortality from dehydration and reduce the treatment seeking costs for caretakers.

### Methodological considerations

Migration was high in the study area. This was mitigated by accounting for dropouts in the sample size calculation. Another limitation was that all information collected was based on caretakers' reports. This method is prone to both recall bias and reporting bias, as the caretaker could have forgotten certain information or reported what they considered was expected of them, respectively. Recall bias was minimized by asking common symptoms, which the caretakers were knowledgeable about, and only those caretakers who were in constant care of the children were the respondents. The presence of the child during the interview was also to assist the caretaker to recall the illness episode. Reporting bias was minimized by using the field assistants, who routinely collect data for the DSS, and these had already established rapport with the respondents during their earlier data collection visits and are knowledgeable on the local situation. Reasons specified for using a provider could refer to reasons for using provider type like drug shop instead of facility or one drug shop instead of another. Because of this, in the bivariate and multivariable analysis, only those conditions that were possible in both the drug shops/private clinics and the government facilities were considered. Choosing one sick child randomly from a family where more than one child was sick could have led to an over representation of households with more than one child since they have more chances of having sick children. Choosing a provider is influenced by the availability of providers in the area. Drug shops and private clinics in the DSS provide similar services and caretakers did not differentiate between the drug shop and a private clinic. In the analysis, drug shops and private clinics were combined and the other option was the government facilities.

## Conclusion

In this study, the majority of caretakers of febrile children under five sought care from drug shops and private clinics, despite the abolition of user fees in government facilities. While the private sector is perceived to provide affordable first aid treatment, the government facilities are thought to offer treatment that is of good quality, but seemingly not affordable to all. Removal of user fees alone will not address the community demand for access to prompt and affordable quality treatment for febrile children. There is need for a multi-pronged approach that would involve free and subsidized drugs, not only from government facilities, but also through private providers and community medicine distributors. Effects on the quality of care children effectively receive through extending the scope of fever treatment to cover malaria, pneumonia and diarrhoea in public as well as private sector should be evaluated.

## Competing interests

The authors declare that they have no competing interests.

## Authors' contributions

ER, GP, SP, GT and KK took part in designing the study, in tools development, in data analysis and in manuscript writing. ER, GP, SP and KK did field work. All authors approved the final manuscript.
